# Evolution of gastroesophageal reflux disease symptoms after bariatric surgery: A dose–response meta-analysis^☆^^☆☆^

**DOI:** 10.1016/j.sopen.2021.11.006

**Published:** 2021-11-19

**Authors:** Abdel-Naser Elzouki, Muhammad-Aamir Waheed, Salah Suwileh, Islam Elzouki, Hisham Swehli, Maryam Alhitmi, Mona Saad, Elmukhtar Habas, Suhail A. Doi, Mohammed I. Danjuma

**Affiliations:** a Department of Medicine, Hamad General Hospital, Hamad Medical Corporation, Doha, Qatar; b Weill Cornell College of Medicine-Qatar; c College of Medicine, Qatar University, Doha, Qatar; d Department of Medicine, Northampton General Hospital, Northampton, UK; e Department of Medicine, Tripoli Central Hospital, Tripoli, Libya

**Keywords:** GERD, gastroesophageal reflux disease, DRMA, dose–response meta-analysis, LSG, laparoscopic sleeve gastrectomy, LRYGB, laparoscopic Roux-en-Y gastric bypass surgery

## Abstract

**Background:**

Obesity is associated with increased prevalence of gastroesophageal reflux disease, with recent reports suggesting improvement in gastroesophageal reflux disease symptoms and weight loss following bariatric surgical intervention. However, the exact impact of the type of bariatric surgery on the evolution of gastroesophageal reflux disease symptoms has remained unexamined.

**Methods:**

We systematically searched electronic databases (PubMed, EMBASE, Web of Science, and the Cochrane Library from inception to December 2018) for eligible studies that satisfy prespecified inclusion criteria. We included clinical trials of all designs that reported on gastroesophageal reflux disease outcomes following laparoscopic sleeve gastrectomy or laparoscopic Roux-en-Y gastric bypass. Two independent reviewers extracted relevant data based on the Preferred Reporting Items for Systematic Reviews and Meta-Analyses guideline. Data were pooled using a random-effects model. Main outcomes were symptomatic improvement in gastroesophageal reflux disease symptoms following bariatric surgery.

**Results:**

A total of 31 studies were analyzed, and a robust-error meta-regression model was used to conduct a dose–response meta-analysis synthesizing data on 31 studies that reported gastroesophageal reflux disease outcomes after bariatric surgery. Of 5,295 patients who underwent either laparoscopic sleeve gastrectomy (*n* = 4,715 patients) or laparoscopic Roux-en-Y gastric bypass (*n* = 580 patients), 63.4% experienced improvement in gastroesophageal reflux disease symptoms (95% CI 32.46–72.18). The dose–response meta-analysis demonstrated a window period of 2 years for sustained improvement after which symptoms began to recur in those that were asymptomatic.

**Conclusion:**

Bariatric surgery may improve gastroesophageal reflux disease symptoms in obese patients who underwent laparoscopic sleeve gastrectomy; however, the most favorable effect is likely to be found after Roux-en-Y gastric bypass surgery. The effects were not sustained and returned to baseline within 4 years.

## INTRODUCTION

The burden of obesity is on the rise across different continents and populations around the world [1]. Uncontrolled obesity has been associated with preventable morbidities across a wide range of cardiovascular and metabolic risks; these include cardiovascular disease, hypertension, diabetes mellitus, venous thromboembolic disease, obstructive sleep apnea, and cancer among others [2]. For most patients, bariatric surgery remains the only option for the treatment of obesity when dietary interventions and pharmacotherapy fail. The range of bariatric surgical options currently in use includes laparoscopic sleeve gastrectomy (LSG) and laparoscopic Roux-en-Y gastric bypass (LRYGB), both of which have been shown to result in varying degrees of weight loss [3]. Gastroesophageal reflux disease (GERD) in particular has been the subject of recent concerns [4]. The prevalence of clinically relevant GERD associated with bariatric surgery is variable but has been reported as ranging between 45% and 50% [[5], [6], [7],^23^]. Uncertainty however remains regarding the exact evolution of symptoms especially among patients with different clinical phenotypes such as metabolic syndrome, obesity, and multimorbidities. In this dose–response meta-analysis (DRMA), we therefore intend to assess GERD symptoms over time with the view to clarify the timeframe of evolution of GERD symptoms among patients that have undergone these procedures**.**

## MATERIALS AND METHODS

### Database Search

Literature search strategies was developed using medical subject headings and text key words related to bariatric surgery and GERD. The specific search strategies were created by a health sciences librarian with expertise in systematic reviews. Following the recommendations of the Preferred Reporting Items for Systematic Reviews and Meta-Analyses statement [8], a systematic literature search was performed using PubMed, Medline, EMBASE, Web of Science, and the Cochrane Library from inception to December 2018 combining the key words *obesity, high BMI, weight loss and gastroesophageal reflux with bariatric surgery OR LSG OR laparoscopic sleeve gastrectomy OR sleeve gastrectomy OR SG OR LRYGB OR laparoscopic Roux-en-Y gastric bypass OR gastric bypass OR GB*. Moreover, we individually observed the reference lists of the selected articles to find other potentially relevant studies. GERD was used as the observation index of outcome after all types of bariatric surgery (ie, improvement of GERD after bariatric surgery or/and the number of cases of new onset or worsened GERD after bariatric procedure). The means and measures of dispersion were approximated from the figures given in the reports.

### Selection of Studies (Inclusion and Exclusion Criteria)

Only single-center reports were included for this meta-analysis. The inclusion criteria were as follows: (1) studies reporting on the efficacy of LSG and/or LRYGB on GERD symptomatology, (2) new onset or worsening GERD after LSG and/or LRYGB, and (3) single center case series. The exclusion criteria include (1) studies with incomplete data for GERD symptoms following bariatric surgery, (2) case reports, and (3) studies reporting in languages other than English. Only the most relevant and comprehensive publications were included in the analysis to avoid duplicates and ambiguity; studies involving nonhuman subjects or papers reporting data from the same study populations were excluded.

### Data Extraction

A team of 2 investigators extracted the data using a standardized form, which was reviewed individually by a third investigator following analysis individually and in duplicate (Table 1). To ensure standardization across the reviewers, a calibration exercise was carried out before starting the actual review. Any disagreement between reviewers was resolved by the consensus of 2 authors and the third reviewer. These indicators included study author/year, study design, level of evidence, sample size, follow-up period, type of bariatric surgery (LSG or LRYGB), GERD improvement rate, method of GERD evaluation, and study country.

**Table 1:** Demographic and clinical characteristics of the included studies

*Author, year*	*Surgery type*	*Sample size*	*Patient with GERD*	*GERD improved (*N*)*	*GERD not improved (*N*)*	*GERD Evaluation Method(s)*	*Follow-up (mo)*	*Study design*	*Country*
Lakdawala, 2010	LSG	50	2	2	0	Symptoms	12	Retrospective	India
	LRYGB	50	6	6	0				
Langer, 2010	LRYGB	3	3	3	0	24 h pH manometry	14	Retrospective	Austria
Omana, 2010	LSG	49	9	2	7	Symptoms	27	Retrospective	USA
	LRYGB	74	9	3	6				
Tai, 2011	LSG	47	8	3	5	Questionnaire	12	Prospective	Taiwan
Gluck, 2011	LSG	204	113	112	1	Questionnaire	36	Retrospective	USA
Chopra, 2012	LSG	174	24	11	13	Symptoms & endoscopy	6	Retrospective	USA
Ekelund, 2012	LRYGB	5	5	5	0	Endoscopy & 24 h pH manometry	1.5	Prospective	Sweden
Carabotti, 2013	LSG	74	20	13	7	Questionnaire	47	Retrospective	Italy
Catheline, 2013	LSG	45	5	0	5	Symptoms & endoscopy	60	Retrospective	France
Daes, 2013	LSG	382	170	160	10	Symptoms & endoscopy	22	Prospective	Colombia
Sharma 2014	LSG	32	8	5	3	Questionnaire	12	Prospective	India
Burgerhart, 2014	LSG	20	14	6	8	Questionnaire, 24 h pH manometry	3	Prospective	Netherlands
Kular, 2014	LSG	76	6	2	4	Symptoms	60	Retrospective	India
	LRYGB	72	5	3	2				
Boza, 2014	LSG	161	7	0	7	Questionnaire	60	Retrospective	Chile
Sheppard, 2015	LSG	205	44	3	41	Symptoms	12	Retrospective	Canada
	LRYGB	173	30	15	15				
Gorodner, 2015	LSG	14	4	0	4	24 h pH manometry	12	Prospective	Argentina
Albanopoulus, 2016	LSG	88	24	16	8	Symptoms & endoscopy	36	Retrospective	Greece
Hendricks, 2016	LSG	919	13	0	13	Symptoms, endoscopy, 24 h pH manometry	78	Retrospective	USA
Casella, 2016	LSG	148	27	19	8	Symptoms & endoscopy	72	Retrospective	Italy
Angrisani, 2016	LSG	105	26	15	11	Symptoms & endoscopy	60	Retrospective	Italy
Aridi, 2016	LSG	76	17	8	9	Symptoms	60	Retrospective	Lebanon
Arman, 2016	LSG	65	7	0	7	Symptoms	153	Retrospective	Belgium
Parmar, 2017	LRYGB	22	10	10	0	Symptoms	24	Prospective	UK
Garg, 2017	LRYGB	40	7	7	0	Questionnaire	24	Retrospective	USA
	LSG	40	4	2	2				
Billing, 2017	LSG	916	142	76	66	Questionnaire	12	Retrospective	USA
Chuffart, 2017	LRYGB	7	2	2	0	Symptoms & endoscopy	72	Retrospective	France
	LSG	41	13	4	9				
Goldenshluger, 2017	LSG	178	28	17	11	Symptoms	36	Retrospective	Israel
Borovicka, 2017	LRYGB	134	48	17	31	Endoscopy & manometry	7.5	Prospective	Switzerland
Kowalewski, 2017	LSG	100	4	0	4	Symptoms	96	Retrospective	Poland
Berry, 2018	LSG	477	121	78	43	Symptoms & endoscopy	36	Retrospective	Chile
Coupaye, 2018	LSG	47	16	10	6	24 h pH manometry	12	Prospective	France

*GERD*, gastroesophageal reflux disease; *LSG*, laparoscopic sleeve gastrectomy; *LRYGB*, laparoscopic Roux-en-Y gastric bypass surgery; *USA*, United State of America; *UK*, United Kingdom.

### Quality Assessment

The methodological quality of clinical trials was assessed by Cochrane Collaboration's tool for assessing risk bias which covers sequence generation, allocation concealment, blinding, incomplete outcome data (eg, dropouts and withdrawals), and selective outcome reporting [9]. The observational studies were assessed using the Newcastle–Ottawa Quality Assessment Scale checklist [10].

### Statistical Analysis

The effect size of interest was the difference in prevalence of GERD symptoms in patients immediately before and at any time point after bariatric surgery reported in the papers. This was modeled as a risk difference in a DRMA. The latter was conducted as a 1-step procedure with time (as the "dose") along with change in risk as the outcome were fit into an inverse variance weighted nonlinear robust error meta-regression model [11], using restricted cubic splines with 3 knots in an effort to approximate the potential nonlinear relationship. In simple terms, this method is a weighted regression model with time since surgery on the *x* axis and change in prevalence of GERD symptoms on the *y* axis so that the trend over time is synthesized. We judge clinical significance from the extent of change in prevalence over time. The weights were based on the inverse of the variance of the incremental risk difference in proportions of patients with GERD symptoms from the baseline at surgery. Stata MP 15 (StataCorp, College Station, TX) was used for the analysis utilizing the *remr* package [12]. Confidence level was set at 95%.

## RESULTS

### Study Features

**Fig 1:**
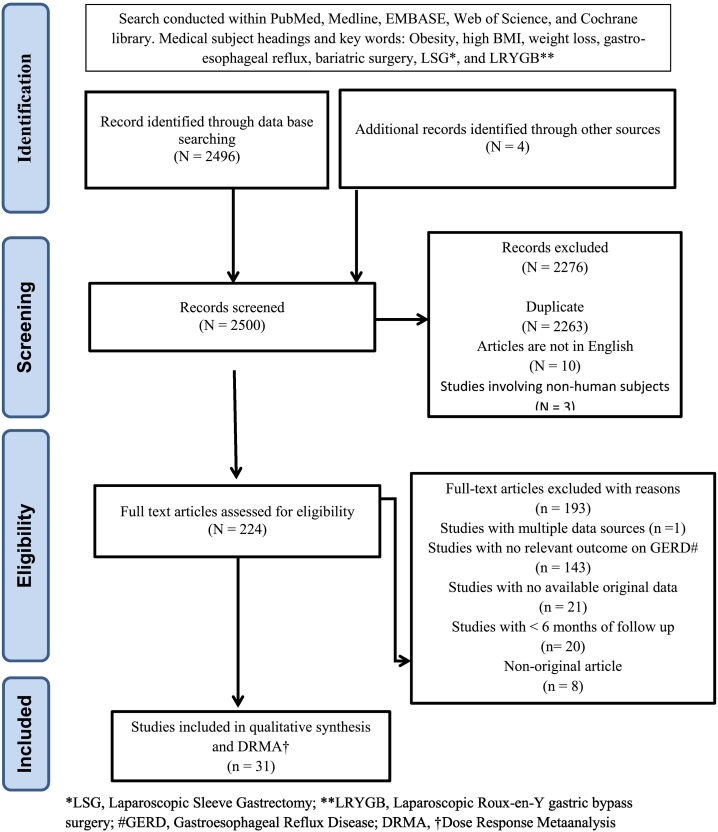
Flowchart of literature search.

A total of 31 studies including 5,295 patients (4,715 in LSG and in 580 LRYGB) were enrolled for the DRMA from a cluster of 2,500 studies pooled from all databases. Figure 1 shows the flowchart of study selection with details of retrieval process and filtering. The 31 selected studies included 9 prospective studies and 23 retrospective observational studies [[13], [14], [15], [16], [17], [18], [19], [20], [21], [22], [23], [24], [25], [26], [27], [28], [29], [30], [31], [32], [33], [34], [35], [36], [37], [38], [39], [40], [41], [42], [43], [44]]. They were conducted in 19 different countries (Argentina, Austria, Belgium, Canada, Chile, Colombia, France, Greece, India, Israel, Italy, Poland, Lebanon, Netherlands, Sweden, Switzerland, Taiwan, United Kingdom, USA) with clinical follow-up of 19.4 (SD ± 31.0) months after bariatric surgery. The demographic and clinical characteristics of the included studies are shown in Table 1.

### Effect of Bariatric Surgery on GERD

**Fig 2:**
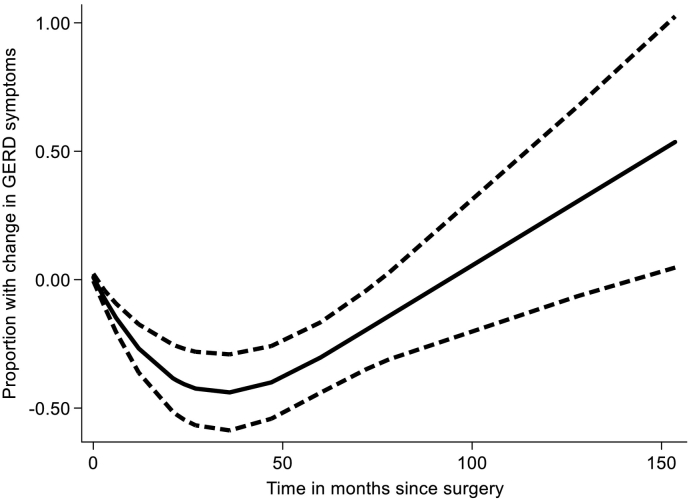
Laparoscopic sleeve gastrectomy (LSG) DRMA results. The figure depicts difference in prevalence from baseline over time of GERD symptoms. Dashed lines depict the 95% confidence intervals.

**Fig 3:**
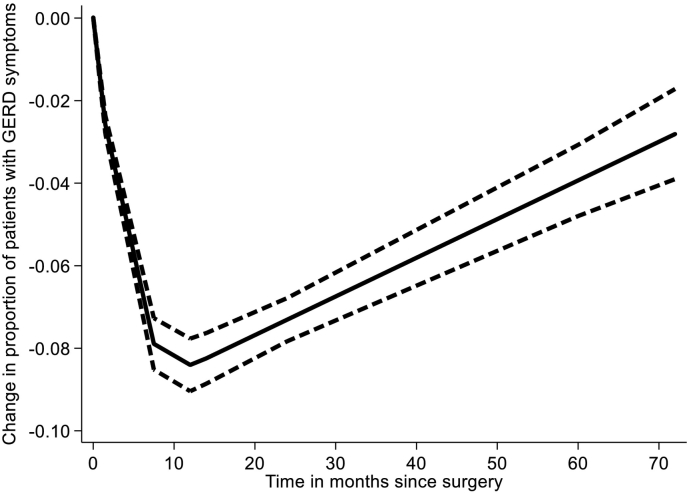
Laparoscopic Roux-en-Y gastric bypass (LRYGB) DRMA results. The figure depicts difference in prevalence from baseline over time of GERD symptoms. Dashed lines depict the 95% confidence intervals.

A total of 31 studies (80.6%) showed improvement or remission for obesity-related GERD (77.7% in LSG, 100% in LRYGB). The proportion of subjects with GERD symptoms was 18.9% (*n* = 1,001 patients, IQR 14%–34%) at baseline and 8.8% (IQR 2.8%–13.6%) at follow-up. Although this seems to suggest a decrease in GERD symptoms after bariatric surgery, the follow-up varied considerably with a median of 24 months (IQR 12–60 months). When the incremental risk was modeled in a DRMA with time as the "dose," there was a decrease in prevalence of GERD symptoms until 2 years postsurgery, and this gradually increased back to baseline at 4 years post-LSG surgery (Fig 2) and post-LRYGB surgery (Fig 3). The trend was a continuing increase in risk, but the trend after 4 years was not reliable owing to a paucity of data points. There was a much greater decrease after LRYGB compared to LSG, but this was driven by a single large study (23), and after this was excluded, the trends were similar. In addition, there were fewer data points for LRYGB.

## DISCUSSION

This meta-analysis represents the first comprehensive attempt at exploring the comparative prevalence of GERD symptoms following either LSG or LRYGB surgery. We found a significant decrease in GERD-related symptoms up to 2 years following surgery. This symptomatic improvement was regardless of the type of surgery. Beyond 2 years however, uncertainty remains as to the impact of surgery on GERD-related symptom relief. The rate of symptomatic improvement was steeper and relapse of symptoms was quicker with LRYGB surgery compared with LSG procedures or be it with an uncertain point estimates. This has significant implication for patient counseling before surgery as well commissioning of surgical procedures. The exact mechanism underpinning the improvement in GERD symptoms following surgery continues to generate intense mechanistic debate. Several reports have attributed this to significant and durable weight reduction evident in patients who underwent these procedures [45,46].

We found an unusually high prevalence of GERD symptoms at baseline before surgery. Previous reports have estimated this at 45%–50% [23,47]. The difference in estimate from our review and that reported from previous studies may have to do with marked heterogeneity in the modalities employed to evaluate GERD. The change or improvement in GERD symptoms (ΔGERD) from 23% at baseline to 8.8% over a median period of 2 years is comparable or better than that seen with antisecretory therapy [48].

The lack of difference in point estimates of residual GERD outcomes between SLG and LRYGB mirrors previous uncertainty regarding the exact impact of the type of bariatric surgery on GERD symptom improvement. The findings from the largest systematic review by Stenard et al exploring this uncertainty in patients who underwent LSG were discordant [49]. About half of the studies in that review found symptomatic improvement in GERD symptoms following the procedure, with the remaining half showing signs of worsening GERD symptomatology. The apparent discordance in GERD outcomes in this review was attributable to heterogeneity in the mode of evaluation of GERD ranging from clinical evaluation, 24-hour ambulatory pH studies, esophageal manometry, or contrast studies, to endoscopy. Additionally, only 1 study was prospective; the remaining studies were retrospective. Other subsequent reviews [50,51] including that by Himpens et al [52] also reported mixed outcomes regarding GERD symptom improvement following LSG. Our review similarly found lack of superiority between different surgical modalities in GERD symptom improvement following surgery. The confounding issues highlighted earlier including marked heterogeneity in the mode of evaluation of GERD may have accounted for our point estimates with regard to effect of bariatric surgical modality on GERD symptom improvement.

Additionally, we found improvement in GERD symptoms plateauing at 4 years with significant uncertainty afterward. Although this suggests the need for more comprehensive studies to ascertain the exact impact of bariatric surgery in GERD symptoms beyond 4 years, it is probable that rapid weight gain reported from previous series may be the key driver to this phenomenon.

### Strengths and Limitation

This DRMA represents the first comprehensive attempt at exploring the comparative efficacy of LRYGB versus LSG surgical procedures in achieving sustained and durable improvement in GERD symptoms. It provides the first estimates of the average duration of GERD symptom improvement (2 years) following these procedures as well as raises the prospects for further studies to explore determinants of uncertainty in symptom relief after 4 years. As with previous systematic synthesis in this area, our DRMA is limited by differences in the modes of adjudication of GERD symptoms by various investigators. The reliance on clinical evaluation alone in some cases and paucity of data regarding utility of PH monitoring as well as endoscopy data in others may have accounted for the imprecision regarding some point estimates in previous studies (including ours).

In conclusion, in obese bariatric patients, we found significant improvement in GERD symptoms at 2 years regardless of the type of bariatric surgery (LSG or LRYGB), but this is not sustained beyond 4 years. The rate of improvement in GERD symptoms was faster but less durable with LRYGB compared to gastric sleeve surgery.

## Author Contribution

A-NE: Review conceptualization, protocol registration, independent reviewer, risk of bias assessment, data interpretation, data interpretation, and writing of the initial draft/revision of the final manuscript.

MID: Risk of bias assessment, data interpretation, writing of the initial draft/revision of the final manuscript.

SD: Statistical data analysis, data interpretation, review of the initial and final manuscript drafts.

M-AW: Protocol registration, independent reviewer, data collection and curation.

SS: Independent reviewer, data collection and curation.

IE: Data collection, revision of final manuscript.

EH: Revision of the final manuscript.

HS: Revision of final manuscript.

MA: Revision of final manuscript.

MS: Revision of final manuscript.

## Conflict of Interest

None of the authors have any conflict of interest to declare.

## Funding Source

Open access funding provided by Qatar National Library with ethical standards.

## Statement of Ethics Approval

As a review and meta-analysis, this article is exempt from mandatory ethics approval.
